# Incentivizing COVID-19 vaccination among racial/ethnic minority adults in the United States: $209 per dose could convince the hesitant

**DOI:** 10.1186/s13561-023-00417-y

**Published:** 2023-01-11

**Authors:** Kevin Chen, Marta Wilson-Barthes, Jeffrey E. Harris, Omar Galárraga

**Affiliations:** 1 Warren Alpert Medical School of Brown University, Providence, RI 02903 USA; 2 Department of Epidemiology, Brown University School of Public Health, Providence, RI 02912 USA; 3 Department of Economics, Massachusetts Institute of Technology, Cambridge, MA 02142 USA; 4 Department of Health Services, Policy and Practice, Brown University School of Public Health, Providence, RI 02912 USA

**Keywords:** Financial incentives, Vaccination, COVID-19, Contingent valuation, Willingness-to-accept, United States

## Abstract

**Background:**

More than two years into the coronavirus disease (COVID-19) pandemic, it remains unclear whether financial incentives can reduce vaccine hesitancy and improve uptake among key unvaccinated populations. This study estimated the willingness of racial/ethnic minority adults in the United States to accept financial incentives for COVID-19 vaccination and the minimum amount needed to vaccinate a sufficiently high percentage of this population.

**Methods:**

From August through September 2021, we conducted an online survey of 367 Black/African American and Hispanic patients, age ≥ 18 years, from 8 community health centers in Rhode Island. Contingent valuation questions assessed respondents’ willingness-to-accept (WTA) incentives for COVID-19 vaccination using random-starting-points and iterative incentive offers of $5 to $50 per dose. Ordered logistic regression models examined associations between respondent characteristics and WTA. Predictive probabilities were modeled using both within-survey range and out-of-survey range incentive offer amounts and compared against vaccination thresholds needed to reach herd immunity.

**Results:**

Less than 30% of unvaccinated survey respondents were WTA an incentive of $50/dose for vaccination. Models using out-of-survey incentive offer amounts greater than $50 suggested that 85% of respondents would agree $140/dose (95% CI: $43-$236) could convince other people to accept vaccination, while $209/dose (95% CI: -$91-$509) would be needed for 85% of respondents to accept vaccination themselves.

**Conclusions:**

Findings from this analysis may inform the design of incentive schemes aiming to reduce racial/ethnic disparities in vaccine and booster uptake, which will continue to be important as new variants of SARS-CoV-2 emerge.

**Supplementary Information:**

The online version contains supplementary material available at 10.1186/s13561-023-00417-y.

## Key points for health policy planners

- Findings from this contingent valuation survey among racial/ethnic minority adults in Rhode Island found that offering $50 per dose was insufficient to incentivize a sufficiently high percentage (≥ 85%) of unvaccinated individuals to accept vaccination against COVID-19.
- Modelled theoretical estimates suggest that at least 85% of racial/ethnic minority adults would be willing to get vaccinated if they received compensation in the range of $140 to $209 per dose.
- Native English-speakers and those who had already received at least one dose of a COVID-19 vaccine were willing to accept a lower incentive amount in exchange for COVID-19 vaccination.

## Background

In the United States (U.S.), unvaccinated adults are approximately 10 times more likely to be hospitalized and 11 times more likely to die of SARS-CoV-2 infection compared to fully vaccinated individuals [1–3]. Yet as of April 2022, nearly 25% of the total U.S. population had not yet received a single dose of a COVID-19 vaccine with younger age, lower education and income, and Republican-leaning political affiliation continuing to be key predictors of vaccine refusal [4, 5]. While racial/ethnic disparities in vaccination rates have narrowed over the course of vaccine rollout for Hispanic adults, Black/African American race/ethnicity continues to be a leading predictor of vaccine hesitancy across geographies and socioeconomic status [6]. As of July, 2022, the CDC reports that race/ethnicity was known for 75% of people who have received one dose of the vaccine. Black/African American people comprise 10% of this group, which is smaller than their share of the total population (12%), and Hispanic people comprise 21% compared to their proportion of the total population (19%). White people made up 55% of those with at least one dose compared to their share of the total population (59%) [6]. The percentage of fully vaccinated Black/African American and Hispanic adults who have received at least one booster dose is also disproportionately lower compared to White adults [6].

During the early stages of the COVID-19 pandemic, state governments as well as public and private employers implemented incentivizing programs, including lotteries and conditional incentives, to encourage COVID-19 vaccination and counteract a persistent critical mass of unvaccinated individuals in the U.S [7–9]. Yet most lotteries and incentive schemes were implemented ad hoc and demonstrated minimal effect on vaccine uptake. For example, a randomized controlled trial offering moderate incentives of $10-$50 to more than 2500 Medicaid patients found no statistically significant improvements in 30-day vaccination rates following incentivization, with similar studies also showing limited improvements in vaccination rates following modest incentives equivalent to $25–$68 per person [10–12]. In Ohio and other states, high-stakes lotteries in which vaccinated winners can earn upwards of $1 million dollars have also failed to significantly increase rates of COVID-19 vaccination or slow declines in daily vaccination rates, compared to trends in non–lottery states [13–16]. Recent commentary suggests that vaccine promotion interventions that offer guaranteed cash payments are more effective and valued by participants, but that persistent gaps in understanding regarding the optimal incentive amount and recipient profile remain [17, 18]. Thus, determining the dollar amount needed to tip the scale for key unvaccinated populations will be a critical step in slowing SARS-CoV-2 transmission and reaching herd immunity thresholds [19–21].

To the best of our knowledge, conditional economic incentives for COVID-19 vaccination have not yet been tested specifically for unvaccinated Black/African American and Hispanic adult populations in the U.S. This study aimed to: 1) estimate the optimal amount needed to incentivize a sufficiently high percentage of Black/African American and Hispanic adults to accept vaccination against COVID-19; and 2) identify key factors associated with vaccine acceptance in these populations. Findings from this pilot study can inform the design of real-life financial incentive programs aiming to encourage vaccination among racial/ethnic minority populations in the United States, which will continue to be important as new variants of SARS-CoV-2 emerge [22].

## Methods

### Study population and data collection

Data were collected via an electronic contingent valuation survey of adults, aged 18 years and older, who self-identified as Black/African American or Hispanic and were active patients of 8 Providence Community Health Centers (PCHC) in Rhode Island. The survey was administered during August and September 2021 via an existing Health Insurance Portability and Accountability Act (HIPAA)-compliant text message system that PCHC uses to communicate with patients. Text message invitations were sent to a convenience sample of eligible patients from each health center for which phone numbers and race/ethnicity information were available. Interested patients confirmed their eligibility electronically, provided electronic informed consent, and were then sent a link to complete the electronic survey. Per the request of PCHC, the survey was limited to 8 questions to prevent response fatigue. Questions were intended to capture personal sociodemographic information, vaccination status, and contingent valuation of hypothetical dollar amounts offered to incentivize people to be vaccinated against COVID-19. The survey took 10 min to complete on average, and all responses remained anonymous. Participants received a $10 electronic Amazon gift card for survey completion. Participants could choose to complete the survey in either English or Spanish.

The survey was designed by researchers at Brown University’s Alpert School of Medicine and School of Public Health and administered by PCHC collaborators. The study was reviewed by the Brown University Human Research Protection Program and received an Exempt Determination on May 6, 2021 (Protocol #2104002981).

### Design of the survey experiment

**Fig. 1:**
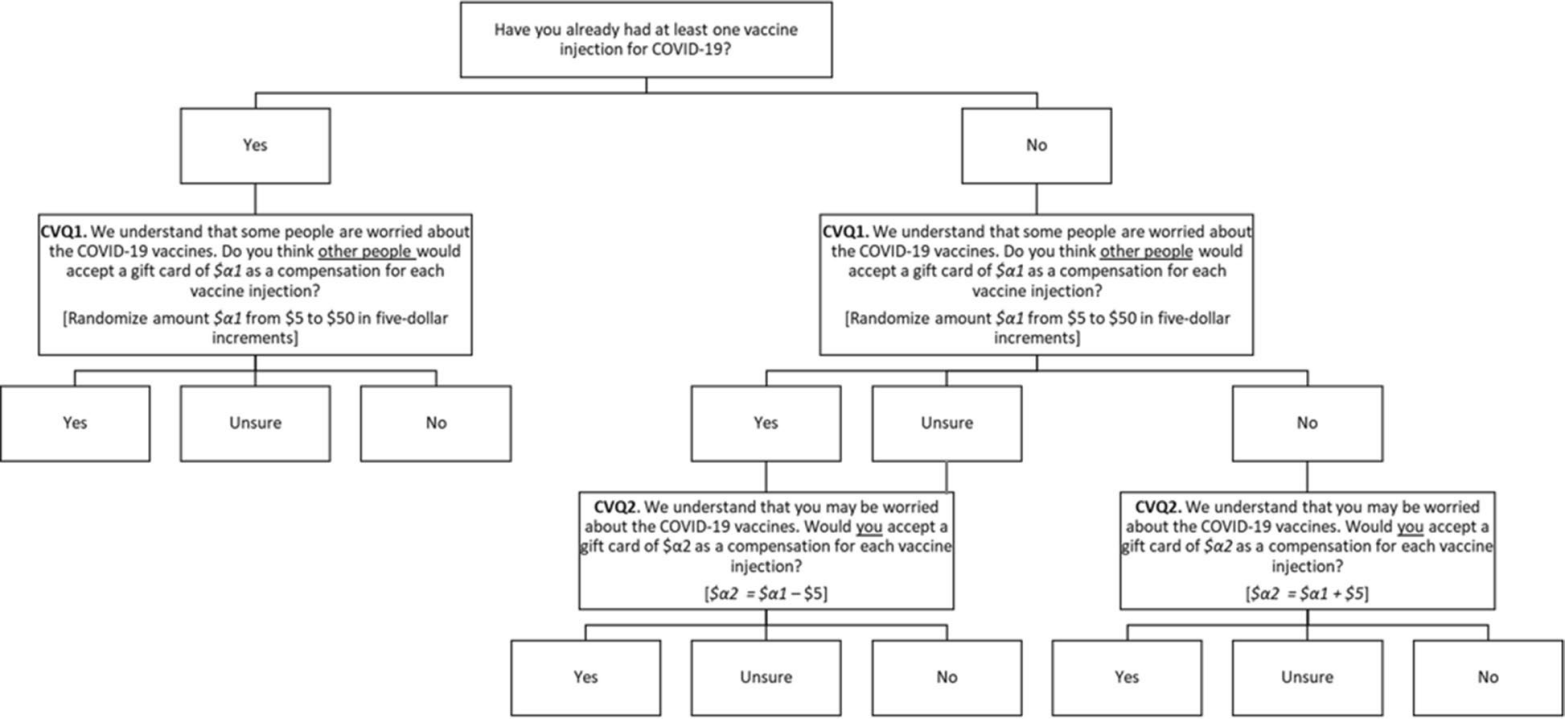
Contingent valuation survey design

We used an embedded survey experiment designed to generate exogenous variation in responses in order to estimate the minimum incentive amount necessary for patients to accept at least one dose of a COVID-19 vaccine [23]. Figure 1 We used random-starting-points and iterative bidding. The computer selected a random incentive amount *$α1* from a predetermined range (i.e., from $5–$50 in $5 increments). The first question asked all respondents if they had received at least one dose of a COVID-19 vaccine at the time of the survey followed by: “Do you think other people would accept a gift card of *$α1* as a compensation for each vaccine injection?”. Only participants who had not received at least one dose of a COVID-19 vaccine at the time of survey completion were then asked a follow-up question: “Would you accept a gift card of *$α2* as a compensation for each vaccine injection?”. The follow-up question raised the *$α2* incentive amount by $5 if the respondent did not accept the first *$α1* amount, and lowered the *$α2* incentive amount by $5 if the respondent was willing to accept or was unsure if they would accept the first *$α1* amount. Response options for each closed-ended contingent valuation question were mutually exclusive (yes/unsure/no). The full survey instrument is provided in Supplementary Information File 1 (SI1).

### Statistical analyses

All analyses were conducted using StataSE 15 software (Stat Corp., College Station, TX, USA). First, for the variables of interest, we reported mean values and standard deviations or frequencies and percentages for continuous and categorical characteristic overall and by vaccination status of the survey respondent. For continuous variables, an ANOVA significance test was used to compare characteristics between vaccinated and unvaccinated individuals. A Pearson’s chi-squared significance test was used for categorical variables. There were no missing data in the final analytic sample.

Second, a multivariate analysis was conducted on the basis of a proportional-odds ordered logit (polytomous logistic) regression model. The dependent variables, i.e., (1) participant’s response to whether other people would be WTA the incentive offer, and (2) participant’s response to whether they themselves would be WTA the incentive offer, were categorized into three graded levels: 0 = No, 1 = Unsure, 2 = Yes, where “Unsure” responses were assumed to express a preference somewhere between “No” and “Yes”. The independent variable was the random *$α1* or contingent *$α2* incentive amount. Selecting the model with the lowest Akaike Information Criterion (AIC) value, all sociodemographic variables captured via the survey were included in the final model as explanatory variables, specifically respondent’s age category (18–29 years; 30–49 years; 50–64 years; 65 years and older), race/ethnicity (Both Black & Hispanic; Hispanic only; Black only), preferred language (English; Spanish), and gender. Gender was initially ascertained using a 7-category question with the following response options: Gender Non-Conforming/Genderqueer; Man; Non-Binary; Transgender Man/Trans Man; Transgender Woman/Trans Woman; Woman; Prefer not to answer. A binary dummy variable for gender was used during analyses (Female or Transgender Woman/Trans Woman = yes/no). The level of statistical significance was set at 0.10. For each WTA estimate, the model provided regression coefficients, the antilogs of which were the odds ratios (OR) expressing the effect of a 1-unit increment of one of the independent variables with all others remaining constant. Specifications of the ordered logit models are included in Supplementary Information File 2 (SI2).

Third, we used Stata’s margins command following the ordered logit regressions to predict the incremental effect of a one-unit ($5) change on the probability that a respondent affirmed others or they themselves would be WTA (WTA = Yes) the theoretical incentive offer for vaccination [24]. Predictive margins were applied using both hypothetical incentive amounts from the contingent valuation survey (i.e., amounts ranging from USD$5–$50 in $5 increments) and out-of-survey amounts ranging from $55 to $250 per dose. For this analysis we use the term “out-of-survey amounts” to refer to theoretical incentive amounts that were modelled but not included in the contingent valuation administered to participants. Predicted probabilities were compared against a threshold of 85% based on the upper bound of commonly cited immunization rates needed to reach herd immunity in the entire U.S. population [20, 21]. Incentive amounts for which the probability of WTA was 0.85, standard errors and 95% confidence intervals were calculated using the Delta method [25, 26].

## Results

From June through July 2021 a total of 7,157 invitation texts were sent to PCHC patients. Of these, 367 patients fully completed the contingent valuation survey and were included in the analytic sample, representing a response rate of about 5%. Table 1 Fifty-two percent of survey respondents self-identified as Black/African American and fifty-one percent self-identified as Hispanic. Respondents were majority female or transgender female (76.8%), under 50 years of age (79.3%), and English-speaking (80.7%). Nearly 72% of respondents had received at least one dose of a COVID-19 vaccine at the time of survey completion. Among the 104 unvaccinated survey respondents, the most commonly cited reason for delaying vaccination was wanting to wait for additional evidence of vaccine safety.

**Table 1:** Descriptive statistics of racial/ethnicity minority adults in Rhode Island who completed the contingent valuation survey

	**All Survey Respondents** **(*N* = 367)**	**Vaccination Status of Respondent at Time of Survey Completion**		***P*-value**
		**Unvaccinated** **(*N* = 104)**	**Vaccinated** **(*N* = 263)**	
**Preferred Language**				
English	296 (80.7%)	91 (87.5%)	205 (77.9%)	0.04
Spanish	71 (19.3%)	13 (12.5%)	58 (22.1%)	
**Age in years**				
18–29	142 (38.7%)	48 (46.2%)	94 (35.7%)	0.18
30–49	149 (40.6%)	40 (38.5%)	109 (41.4%)	
50–64	66 (18.0%)	15 (14.4%)	51 (19.4%)	
65 +	10 (2.7%)	1 (1.0%)	9 (3.4%)	
**Gender**				
Female / Transgender Female	282 (76.8%)	86 (82.7%)	196 (74.5%)	0.05
Male / Transgender Male	82 (22.3%)	16 (15.4%)	66 (25.1%)	
Other^a^	3 (0.8%)	2 (1.9%)	1 (0.4%)	
**Black or African American**				
Yes	191 (52.0%)	57 (54.8%)	134 (51.0%)	0.51
No	176 (48.0%)	47 (45.2%)	129 (49.0%)	
**Hispanic**				
Yes	187 (51.0%)	50 (48.1%)	137 (52.1%)	0.49
No	180 (49.0%)	54 (51.9%)	126 (47.9%)	
**Main reason for choosing to not get a COVID-19 vaccine**				
Concerns about rushed timeline	10 (12%)	10 (12%)	-	
Want to wait to confirm the vaccines are safe	25 (30%)	25 (30%)	-	
Don’t trust vaccines generally	12 (15%)	12 (15%)	-	
Want to wait to see how effective the vaccines are	14 (17%)	14 (17%)	-	
Other reason^b^	21 (26%)	21 (26%)		

All demographics characteristics were based on self-report

^a^The “Other” gender category includes survey respondents who selected “Gender Non-Confirming”, “Non-Binary”, or “Prefer not to answer”

The *P*-value indicates whether there was a statistically significant difference at the 0.10 level between vaccinated and unvaccinated survey respondents for the relevant characteristic

^b^The “Other reason” category includes any written-in reason. Examples of other reasons survey respondents cited for not getting vaccinated included “Against my religion”, “Breastfeeding still”, “Fear of needles”, as well as fear of adverse reactions and lack of trust in the pharmaceutical companies

**Fig. 2:**
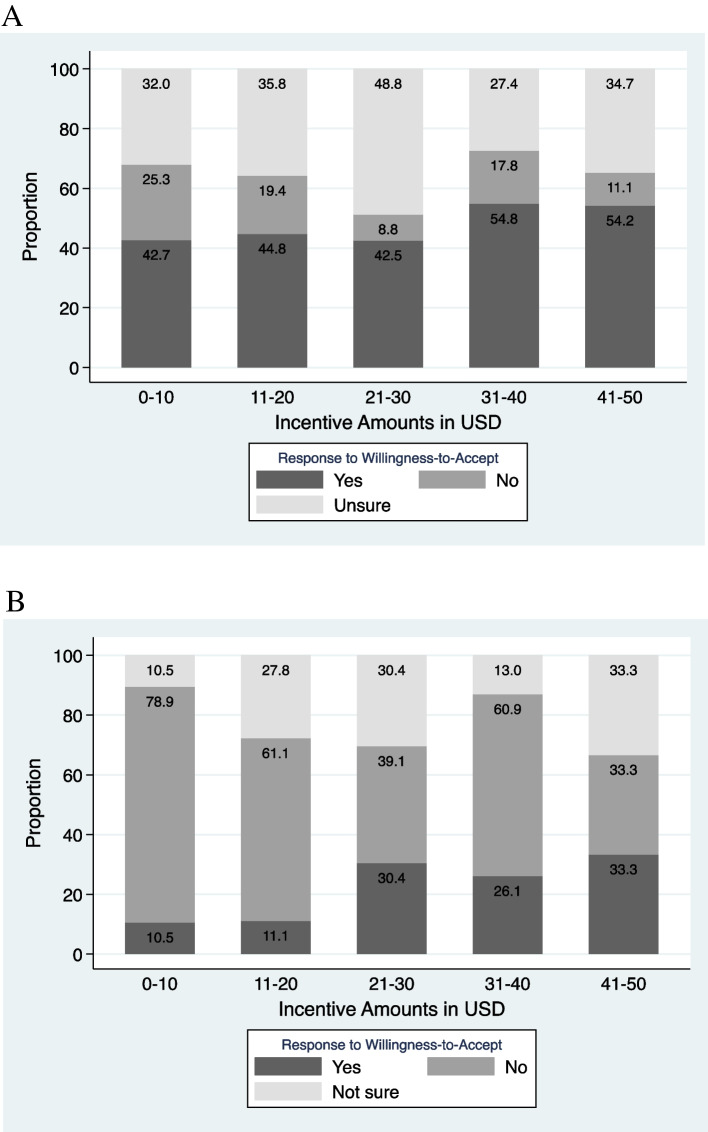
**a** Proportion of “other people” willing to accept conditional economic incentives as compensation for receiving a single dose of a COVID-19 vaccine. Figure shows responses from the 367 individuals who responded to the contingent valuation question: “Do you think other people would accept a gift card of *$α1* as a compensation for each vaccine injection?”, where *$α1* was a randomly generated incentive amount ranging from USD $5—$50. Acceptance variables were 3-level, mutually exclusive categorical responses = 3 if respondent was willing to accept incentive amount, = 2 if unsure, and = 1 if not willing to accept. **b** Proportion of survey respondents willing to accept conditional economic incentives as compensation for receiving a single dose of a COVID-19 vaccine. Figure shows responses from the 104 individuals who responded to the contingent valuation question: “Would you accept a gift card of $*α*2 as a compensation for each vaccine injection?”, where $*α*2 was a randomly generated incentive amount ranging from USD $5—$50. Acceptance variables were 3-level, mutually exclusive categorical responses = 3 if respondent was willing to accept incentive amount, = 2 if unsure, and = 1 if not willing to accept

Figure 2a shows the proportion of survey respondents who selected each possible response (“No”, “Unsure”, Yes”) when considering if other people would be willing to accept COVID-19 vaccination for incentive offers up to USD$50 (*$α1*). The proportion of participants who selected “Yes” increased with increasing incentive amounts. Forty-three percent (42.7%) of participants thought other people would be WTA incentives in the range of 0-$10 for vaccination compared to 54.2% for incentives in the range of $41-$50USD. Figure 2b shows the proportion of survey respondents who selected each possible response when considering if they themselves would be willing to accept COVID-19 vaccination for incentive offers up to USD$50 (*$α2*). Those who selected “Yes” increased with increasing amounts, ranging from 10.5% at incentives between $0-$10 to 33.3% at incentives between $41-$50.

Table 2 presents the results of the ordered logistic regression models analyzing factors associated with willingness to accept incentive amounts that had been offered in the survey for COVID-19 vaccination. In both adjusted and unadjusted models, the odds of other people being willing to accept incentives for vaccination increased by 2% with each $5 increase in incentive amount (odds ratio (OR) and 95% Confidence Interval (CI): 1.02 (1.00–1.03) in the unadjusted model (*p* = 0.039); and odds ratio (aOR) and 95% CI: 1.02 (1.00–1.03) in the adjusted model (*p* = 0.011)). The odds that respondents believed other people would be WTA the random incentive amount was 2.72 times greater for vaccinated compared to unvaccinated respondents (95% CI: 1.82–5.75; p < 0.01) and 3.27 times greater for English-speaking compared to Spanish-speaking respondents (95% CI: 1.73–4.27; p < 0.01). Age, race and gender of the respondent were not statistically significantly associated with believing other people would be WTA the random incentive amount. Odds of the respondents themselves being WTA the incentive were not significantly associated with incentive amount or any other sociodemographic indicator.

**Table 2:** Factors associated with willingness to accept conditional economic incentives to be vaccinated against COVID-19

	**Other Peoples’ WTA** **(1)**		**Respondent’s WTA** **(2)**	
	**OR (95% CI)**	**aOR (95% CI)**	**OR (95% CI)**	**aOR (95% CI)**
**Conditional incentive amount**	1.02 (1.00, 1.03)**	1.02 (1.00, 1.03)**	1.02 (0.99, 1.05)	1.01 (0.98, 1.05)
**Age**^a^	-	1.08 (0.84, 1.39)	-	0.84 (0.49, 1.45)
**Race/Ethnicity**				
Both Black & Hispanic	-	(ref)	-	(ref)
Hispanic only	-	0.80 (0.24, 2.70)	-	1.08 (0.08, 14.43)
Black/African American only	-	0.43 (0.12, 1.46)	-	0.38 (0.03, 4.87)
**Female gender**				
No		(ref)		(ref)
Yes		1.14 (0.71, 1.82)		2.33 (0.72, 7.56)
**Preferred Language**				
Spanish	-	(ref)	-	(ref)
English	-	3.23 (1.82, 5.75)***	-	0.69 (0.21, 2.20)
**Respondent’s vaccination status**^b^				
Unvaccinated	-	(ref)	-	-
Vaccinated	-	2.72 (1.73, 4.27)***	-	-
**Observations**	367	367	104	104
**Pseudo R-squared**	0.006	0.050	0.0065	0.017
**Probability**	0.039	0.000	0.242	0.073
**Log Likelihood**	-371.10	-354.43	-104.37	-97.36

*CI* Confidence Interval, *OR* Odds Ratio, *aOR* Adjusted Odds Ratio, *SE* Standard Error, *WTA* Willingness-To-Accept

^*^p < 0.10, **p < 0.05, ***p < 0.01; Table 2 presents the results from ordered logit regression models

^a^Age was treated as a continuous variable in ordered logit regression analysis such that an odds ratio > 1 indicates a positive association between increasing odds of willingness to accept and being in an older age group

^b^All survey respondents regardless of vaccination status were asked the first contingent valuation question (i.e., how much they thought other people would be WTA for vaccination). Only unvaccinated survey respondents were asked the second contingent valuation question (i.e., how much they themselves would be WTA for vaccination)

**Fig. 3:**
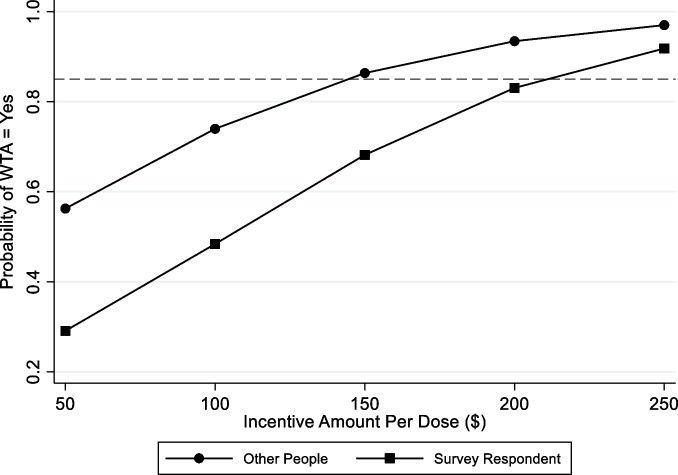
Predictions of the incentive amount racial/ethnic minority adults in Rhode Island would be willing to accept for COVID-19 vaccination. Figure shows the predicted probabilities associated with the incentive amounts needed for others to be willing-to-accept COVID-19 vaccination as well as respondents themselves to be WTA vaccination based on results from the margins post-estimation command. Probabilities were predicted using higher theoretical incentive amounts ranging from USD$50 – $250 per dose, and do not represent WTA probabilities from contingent valuation survey data. Horizontal reference line is set at 85% based on the upper bound of most commonly cited immunization rates needed to reach herd immunity in the entire U.S. population.^20^ WTA: Willingness-To-Accept

Out-of-survey estimates calculated using the incremental effects of the ordered logit regressions indicated that the probability of respondents being willing to accept incentives for vaccination would reach necessary thresholds if incentive amounts were increased to $210 per dose. Figure 3 Using theoretical amounts ranging from $50 to $250 per dose showed that 85% of survey respondents would agree $140 per dose (95% CI: $43—$236) is enough to convince other people to accept vaccination, while $209 per dose (95% CI: -$91—$509) would be needed for 85% of participants to be vaccinated themselves. Figure 4 (Supplementary Information) depicts the predicted probabilities of being WTA incentives for the sociodemographic factors that were statistically associated with odds of being WTA incentives.

## Discussion

This pilot study among patients of community health centers in Rhode Island found that offering $50 per dose was not enough to encourage at least 85% of racial/ethnic minority adults to get vaccinated against COVID-19. On average, increasing the incentive amount by $5 contributed to only minimal (2%) improvements in the odds that this population would be willing to receive a vaccine. Out-of-survey forecasts suggest that $140-$209 per dose could incentivize at least 85% of unvaccinated Black/African American and Hispanic adults to accept vaccination.

Our findings are consistent with recent work in the United States indicating that moderate incentives of $50 or less have minimal impact on improving COVID-19 vaccine uptake. As mentioned previously, the randomized trial of Medicaid patients (NCT04867174) showed that vaccination rates did not significantly improve after 30-days among patients randomized to receive either $10 or $50, compared to non-incentivized controls [10]. Similar effects are seen in other high-income countries, where moderate, monetary incentives of 25 to 50 euros (~ $28 to $57) for vaccination have a much smaller impact on vaccine uptake than increasing vaccine access or offering societal freedoms (e.g., relaxed quarantine requirements) to vaccinated individuals [27]. Conditional lotteries with higher incentive amounts ranging from $68 per participant to over $1 million also have provided insufficiently persuasive evidence as effective tools for increasing state-level vaccination rates, which is unsurprising given that unvaccinated adults in the U.S. seem to prefer guaranteed cash incentives of $100 per dose over lotteries with higher possible winnings [11, 13, 14, 16, 18]. Though North Carolina’s pilot incentive program showed that vaccine initiation declined less at health centers guaranteeing a $25 cash card to adults who received or drove someone to receive their first dose of COVID-19 vaccine compared to facilities without incentives, findings were based on aggregated, clinic-level data such that associations between patient race/ethnicity and vaccination could not be assessed [12]. To our knowledge, the incentive amounts administered by the aforementioned programs were largely chosen arbitrarily and were not based on any formal prior evaluation of patient preferences (e.g., via a discrete choice experiment) or patients’ WTA (e.g., via a contingent valuation survey), which could explain the minimal observed effect. Future incentive programs may be able to increase their impact on vaccine uptake rates if they derive their incentive amounts from patient-centered empirical evidence, including the findings from the present survey.

The mean WTA estimates of $140 to $209 per dose found in this study are lower than similar values reported earlier in the pandemic; data ascertained by Carpio and colleagues from 2000 U.S. adults in December 2020 and January 2021 found that median payments of at least $525 would be needed to sufficiently incentivize 50% of individuals who were willing to get vaccinated only if compensated [28]. However, this $525 incentive amount is similar to our findings were we to assume the upper bound ($500/dose) for respondents’ own WTA. Nevertheless, the difference in WTA values over the last 1.5 years of the pandemic may reflect a growing trust in the COVID-19 vaccines due to longer evidence of vaccine safety and efficacy or improved public health messaging [29]. Additionally, we hypothesize that two biases may have contributed to respondents believing others would be WTA a lower incentive offer than they themselves were WTA. Social desirability bias, or the tendency for respondents to bias their responses to appear more favorable, could have contributed to respondents selecting a lower amount for others’ WTA if they believed this was the researchers’ desired answer. Second, because only unvaccinated individuals were asked the follow-up question, bias conferred by deep-rooted vaccine hesitancy could have led to unvaccinated participants requiring a greater incentive than others to be willing to change their own vaccine-acceptance behavior.

Building on the previously mentioned work, the present study offers novel empirical evidence of the minimal amount needed to incentivize a sufficient percentage of two racial/ethnic populations that have disproportionately faced health disparities during the COVID-19 pandemic. To the best of our knowledge, our study is the first to have elicited perspectives exclusively from Black/African American and/or Hispanic/Latinx respondents. Though prior studies of financial incentives for COVID-19 vaccination have disaggregated analyses by race, focusing the study on Black and Hispanic populations allows for within-group analysis of differences in vaccination. [10, 30]

This study has some limitations. First, patients were required to have a working, text message-enabled phone to be invited to complete the survey; this requirement could have introduced selection bias if there were important socioeconomic or demographic differences between patients who had access to a working cell phone and those who did not. Second, the high levels of non-response as well as the > 75% of respondents being female likely introduced selection bias into our sample. However, community health center patients in the United States are disproportionately female, which is consistent with our surveyed population [31]. Furthermore, as described by Rupp and colleagues, it remains unclear how characteristics of survey respondents compare to those of non-responders; some prior work has found non-respondents to be characterized by worse health compared to respondents, while other research has observed no differences in health status [32]. Other factors such as education level, foreigner status, or limited availability due to employment demands may have further contributed to selection bias and potentially limit the generalizability of our findings [33, 34]. Third, possible discrepancies between stated intent versus actual behavior are inherent to willingness-to-accept analyses [6, 35, 36]. Nonetheless, stated-preference methods are still useful for gauging behavior prospects and the phone-based, self-administered survey design likely helped minimize the potential for social desirability bias [37]. Fourth, because results of logistic regression analyses using in-survey incentive amounts resulted in largely null findings, the estimates of $140 to $209 per dose needed to achieve at least 85% vaccine uptake are based on out-of-survey predictions. Though out-of-survey (out-of-sample) forecasting is commonly used in econometrics, it will be necessary to test these higher incentive levels in a larger contingent valuation survey or randomized trial in order to determine the feasibility and acceptability of these amounts [38, 39]. A larger contingent valuation survey or pilot trial can also help narrow the optimal incentive amount within the $140 to $209 range, which may potentially be cost saving for program administrators. Lastly, results were based on data from 367 community health center Black and Hispanic patients in Rhode Island such that our findings are not necessarily generalizable to Black and Hispanic adults in other parts of the U.S. Due to the available patient populations and vaccine uptake rates in Rhode Island at the time of the survey, we restricted the study population to the racial/ethnic groups at greatest risk for adverse health outcomes including lower vaccine uptake. However, this limited our ability to compare our WTA estimates to White adults and other racial/ethnic minorities, which should be explored in future work. Due to survey length constraints, more detailed demographic data including socioeconomic status, geography and comorbidities were not captured. Future research should capture these additional data as well as political affiliation [40] to more comprehensively identify the key determinants of vaccine acceptance.

Survey data for this study were ascertained prior to the emergence of the highly contagious Omicron variant in the United States and before most adult populations were eligible to receive a vaccine booster dose of an mRNA or Janssen COVID-19 vaccine [41, 42]. It is possible that survey respondents would have been willing to accept lower incentive amounts during periods of peak COVID-transmission or immediately following emergency use authorization of boosters when there may have been a greater “demand” for vaccination. However, it is unlikely that a sufficient percentage of survey respondents would have been WTA incentive amounts as low as $50 per dose given the high WTA values reported at the pandemic’s start and the documented hesitancy of Black individuals specifically to accept vaccination without payment [27]. Also, our choice of the 85% vaccination threshold needed to reach herd immunity in the U.S. was based on the infectiousness of the COVID-19 genotypes circulating at the time of data collection [20, 21]. Applying alternative thresholds based on broader health policy goals for disease control may be more appropriate as population behavior and infectiousness of the virus change over time [21, 43].

Common deterrents to vaccination among racial/ethnic minority populations can center on high mistrust of the vaccine itself (e.g., concerns about harmful side effects) and weak subjective norms for vaccination in one's close social network [44–50]. Optimal incentive levels must therefore be tailored to the specific risk- and vulnerability-profile of these groups, being high enough to encourage positive behavior change but not so high as to exacerbate potential undue influence concerns [51]. And though racial equity in COVID-19 vaccination rates has improved in recent months, continuing to encourage vaccination among racial/ethnic minority populations is necessary to address persistent disparities in the uptake of booster shots as they become available to the general public [6, 52]. Determining appropriate incentive levels *prior to* implementation may increase the likelihood that racial/ethnic minority adults are willing to get vaccinated, and help reduce economic and operational inefficiencies for public health programs.

## Conclusions

This study found that incentives up to $50 per dose are insufficient to persuade a sufficiently high percentage of Black/African American and Hispanic adults to accept COVID-19 vaccination. Offering $140 to $209 per dose could be enough to surpass recommended vaccination thresholds needed to achieve herd immunity in the United States. Further evidence from a randomized trial is needed to inform the effectiveness and cost-effectiveness of offering incentive amounts above the $140-$209 range for vaccine-hesitant populations.

## Supplementary Information

**Additional file 1.**

## Data Availability

The de-identified datasets generated and/or analyzed during the current study and the corresponding code used to conduct the analysis reported in this manuscript are available in the Brown University Library Digital repository, https://doi.org/10.7910/DVN/8HR2LI.

## Abbreviations

HIPPA: Health Insurance Portability and Accountability Act
PCHC: Providence Community Health Centers
WTA: Willingness to Accept
