# Supplementary material for: Incentivizing COVID-19 vaccination among racial/ethnic minority adults in the United States: $209 per dose could convince the hesitant
Source: Health Econ Rev. 2023 Jan 11;13:4. doi: 10.1186/s13561-023-00417-y (PMC9832714; doi:10.1186/s13561-023-00417-y)
Supplement: Supplementary file 1 — Additional file 1. [file 13561_2023_417_MOESM1_ESM.docx]

**Supplementary Information for the manuscript:**

**Full Title:** Incentivizing COVID-19 vaccination among racial/ethnic minority adults in the United States: $209 per dose could convince the hesitant

**Running Title:** Incentivizing COVID-19 vaccination for racial/ethnic minority adults

Contents

[**Supplementary Information – File 1** 3](#_Toc118393530)

[Contingent Valuation Survey – English Version 4](#_Toc118393531)

[Contingent Valuation Survey – Spanish Version 7](#_Toc118393532)

[**Supplementary Information – File 2** 10](#_Toc118393533)

[Specifications of the proportional-odds ordered logit regression models 10](#_Toc118393534)

[**Supplementary Information – Figure 4** 11](#_Toc118393535)

[**References** 12](#_Toc118393536)

# **Supplementary Information – File 1**

## Contingent Valuation Survey – English Version

**Recruitment Text:**

We care about your family’s health and want to know how you feel about the new COVID-19 vaccines now available to patients. The three vaccines that are currently available are highly effective and do not cause any serious side effects. To understand how you feel about the vaccines, we have worked with a Brown University research study [Study Protocol #2981] to create a short survey. The survey will ask you questions about why you would or would not decide to get a COVID-19 vaccine. To be eligible to take the survey, you must self-identify as African-American and/or Latinx and be at least 18 years old.

The survey is 8 questions long and will take 5-10 minutes to complete. Your survey answers will be kept private. We will not give your answers to anyone else. We will not put your answers in your medical record. You can skip any question that you do not want to answer. If you have any questions, you can contact Kevin Chen at [chen_kevin@brown.edu](mailto:chen_kevin@brown.edu) or Omar Galárraga at [omar_galarraga@brown.edu](mailto:omar_galarraga@brown.edu)

You will receive a $10 Amazon Gift Card for completing the survey.

If you are interested in taking the survey, please click on the link below to begin.

**Informed Consent form:**

BROWN UNIVERSITY

CONSENT FOR RESEARCH PARTICIPATION

[Incentives to Vaccinate Against Covid-19 (I-VAC)]

[Version 1, [Study Protocol #2981], 4/18/21]

You are invited to take part in a Brown University research study. Your participation is voluntary.

- RESEARCHER: Omar Galárraga, PhD, Brown University School of Public Health.
- PURPOSE: The study wants to understand if certain groups of people in Rhode Island think that the COVID-19 vaccines are safe and effective. This study also wants to understand if giving economic incentives - such as a small amount of money or a small prize - can help people get vaccinated against COVID-19. You are being asked to be in this study because you are at least 18 years old and you have self-identified as African-American and/or Latinx.
- PROCEDURES: If you choose to participate in this study, you will be asked to complete a short survey on the next page. The survey will ask you questions about why you would or would not decide to get a COVID-19 vaccine. All questions will be optional. You can skip any question that you do not want to answer.
- TIME INVOLVED: The study will take 5-10 minutes of your time.
- COMPENSATION: You will receive a $10 Amazon Gift Card as a thank you for your time.
- RISKS: There is minimal risk from participating in this study. Your answers to the survey questions will remain anonymous. You can skip any question that you do not want to answer. You can stop the survey at any time.
- BENEFITS: There is no direct benefit from being in this research study.
- CONFIDENTIALITY: All data collected by this survey will remain anonymous. To maintain confidentiality, we will assign all your data a coded number. Your responses will not be connected to your identity. Your survey data will be destroyed within 5 years after the study is over. Your anonymous responses may be used for scientific purposes or shared with other researchers for future research, teaching or publication. At the end of the survey, you will be asked to enter your phone number that received the invitation and your email so that we may send you the Amazon Gift Card. Your phone number and email will be entered into a secure dataset that will be destroyed after we deliver the compensation.
- VOLUNTARY: You do not have to be in this study if you do not want to be. Even if you decide to be in this study, you can change your mind and stop at any time.
- CONTACT INFORMATION: If you have any questions about your participation in this study, you can email Kevin Chen at [chen_kevin@brown.edu](mailto:chen_kevin@brown.edu) You can also contact the principal investigator Omar Galarraga, PhD at [omar_galarraga@brown.edu](mailto:omar_galarraga@brown.edu)
- YOUR RIGHTS: If you have questions about your rights as a research participant, you can contact Brown University’s Human Research Protection Program at 401-863-3050 or email them at [IRB@Brown.edu](mailto:IRB@Brown.edu)
- CONSENT TO PARTICIPATE: Clicking the “Yes” option below confirms that you have read and understood the information in this document, are 18 years of age or older and that you agree to volunteer as a research participant for this study.

You can print a copy of this form.

<include URL>

**Electronic survey administered to participants who provided electronic informed consent**

Thanks for your help today!

We care about your family’s health and want to know how you feel about the new COVID-19 vaccines now available to patients. The three vaccines that are currently available are highly effective and do not cause any serious side effects. Please find more information on COVID-19 and advice for the general public that the World Health Organization has put together here: <https://www.who.int/emergencies/diseases/novel-coronavirus-2019/advice-for-public>

We will not give your personal answers to anyone, and we will not put your answers in your medical record.

You can skip any question that you do not want to answer. You can stop the survey at any time.

1. Have you already had at least one vaccine injection for COVID-19?

◉ Yes ◎ No. (If YES, skip to Q4. and then on to Q.6)

2. Do you think you will get the COVID-19 vaccine?

◉ Yes ◎ No ◎ Not Sure (If YES, skip to Q.4)

3. What is the main reason that you would choose not to get a COVID-19 vaccine?

◉ Concerns about rushed timeline

◎ Want to wait to confirm the vaccines are safe

◎ Don’t trust vaccines generally

◎ Want to wait to see how effective the vaccines are

◎ Other reason ____________

4. We understand that some people are worried about the COVID-19 vaccines. Do you think **other people** would accept a gift card of X1 as a compensation for each vaccine injection? [Randomize amount X1 from $5 to $50 in five-dollar increments]

◉ Yes ◎ No ◎ Not Sure

5. We understand that you may be worried about the COVID-19 vaccines. Would **you** accept a gift card of X2 as a compensation for each vaccine injection? [Randomize amount X2 upwards from X1 if answer to previous question (Q4) was no; or randomize amount X2 downwards if answer to previous question (Q4) was yes.]

◉ Yes ◎ No ◎ Not Sure

6. Your age group:

◉ 18-29 ◎ 30-49 ◎ 50-64 ◎ 65+

7. How do you identify yourself:

◉Gender Non-Conforming/Genderqueer

◎Man

◎Non-Binary

◎Transgender Man/Trans Man

◎Transgender Woman/Trans Woman

◎Woman

◎Not listed (please state): _______________________

◎Prefer not to answer

8. How do you identify? Check all that apply:

◉ Black

◎ White

◎ Asian

◎ Native American

◎ Native Hawaiian/Pacific Islander

◎ More than one race

◎ Hispanic / Latinx

◎ Not Hispanic / Latinx

◎ African American

◎ Asian American

◎ Other / Would rather not say

## Contingent Valuation Survey – Spanish Version

**Recruitment text:**

Nos preocupa la salud de su familia. Queremos conocer su opinión sobre las vacunas contra el COVID-19. Las vacunas protegen contra el virus y no provocan efectos secundarios graves. Le invitamos a un estudio de investigación con la Universidad de Brown [Protocolo #2981], para entender lo que Ud. piensa sobre las vacunas a través de una breve encuesta. Estamos invitando a participar a personas que se auto-identifican como afroamerican@s o latin@s y que sean mayores de 18 años.

La encuesta tiene 8 preguntas y dura de 5 a 10 minutos. No daremos sus respuestas personales a nadie, y no pondremos sus respuestas en su expediente médico. Si hay alguna pregunta que lo incomode, puede no responder. Si tiene alguna pregunta, comuníquese con Kevin Chen [[chen_kevin@brown.edu](mailto:chen_kevin@brown.edu)] o con Omar Galárraga [omar_galarraga@brown.edu]

Recibirá una tarjeta de regalo de Amazon de $10 por completar la encuesta. Si está interesad@, haga clic en el enlace a continuación para comenzar la encuesta.

**Informed Consent form:**

UNIVERSIDAD DE BROWN

CONSENTIMIENTO PARA LA PARTICIPACIÓN EN INVESTIGACIÓN

[Título del estudio: Incentivos para Vacunar Contra COVID-19 (I-VAC)]

[Versión 1, [Protocolo #2981], 22/4/21]

Le estamos invitando a participar en un estudio de investigación de la Universidad de Brown. Su participación es voluntaria.

- INVESTIGADOR: Omar Galárraga, PhD, Escuela de Salud Pública de la Universidad de Brown.
- OBJETIVO: Este es un estudio para comprender lo que las poblaciones minoritarias en Rhode Island piensan sobre la vacunación contra el COVID-19 y si los incentivos económicos (como por ejemplo, montos modestos de dinero o pequeños premios) pueden ayudar a la gente a tomar la decisión de recibir la vacuna contra el COVID-19. Le estamos invitando a que participe en este estudio porque Ud. tiene por lo menos 18 años de edad y se ha autoidentificado como afroamerican@ y / o latin@.
- PROCEDIMIENTOS: Si decide participar en este estudio, le pediremos que responda a una breve encuesta en la página siguiente. La encuesta tiene preguntas sobre la decisión de recibir o no la vacuna contra el COVID-19. Todas las preguntas serán opcionales.
- TIEMPO NECESARIO: El estudio tomará de 5 a 10 minutos de su tiempo.
- COMPENSACIÓN: Recibirá una tarjeta de regalo de Amazon de $10 como agradecimiento por su participación.
- RIESGOS: Los riesgos son mínimos en este estudio. Sus respuestas permanecerán anónimas. Puede no contestar cualquiera de las preguntas. Puede parar de contestar la encuesta en cualquier momento.
- BENEFICIOS: No hay un beneficio directo para Ud. al participar en este estudio.
- CONFIDENCIALIDAD: Todos los datos recopilados en esta encuesta permanecerán anónimos. Para mantener la confidencialidad, se asignará a todos sus datos un número codificado. Sus respuestas no estarán conectadas a su identidad. Los datos de su encuesta se destruirán 5 años después de la finalización del estudio. Sus respuestas anónimas pueden usarse con fines científicos o compartirse con otros investigadores para futuras investigaciones, docencia o publicación. Al final de la encuesta, se le solicitará que ingrese su correo electrónico para que podamos enviarle la Tarjeta de regalo de Amazon. Su correo electrónico se ingresará en una base de datos segura que se destruirá después de que le entreguemos su compensación.
- PARTICIPACIÓN VOLUNTARIA: No es necesario que participe en este estudio si no lo desea. Incluso si decide participar en este estudio, puede cambiar de opinión y detenerse en cualquier momento.
- INFORMACIÓN DE CONTACTO: Si tiene alguna pregunta sobre su participación en este estudio, dirija un correo electrónico a Kevin Chen ([chen_kevin@brown.edu](mailto:chen_kevin@brown.edu)). También puede comunicarse con el investigador principal Omar Galárraga, PhD ([omar_galarraga@brown.edu](mailto:omar_galarraga@brown.edu))
- SUS DERECHOS: Si tiene preguntas sobre sus derechos como participante de una investigación, puede comunicarse con el Programa de Protección de Investigaciones Humanas de la Universidad de Brown al 401-863-3050 o enviarles un correo electrónico a [IRB@Brown.edu](mailto:IRB@Brown.edu)
- CONSENTIMIENTO PARA PARTICIPAR: Al hacer clic en el enlace a continuación, confirma que ha leído y comprendido la información de este documento, que Ud. tiene al menos 18 años y acepta voluntariamente ser participante de la investigación en este estudio.

Puede imprimir una copia de este formulario.

<incluir URL>

**Electronic survey administered to participants who provided electronic informed consent**

¡Gracias por su ayuda! Nosotros nos preocupamos por su salud. Nos gustaría saber lo que Ud. piensa sobre las vacunas contra el COVID-19. Las tres vacunas ya aprobadas son muy efectivas y no causan ningún efecto secundario serio.

Hay más información sobre el COVID-19 y consejos de la Organización Mundial de la Salud para el público en este sitio web:

<https://www.who.int/es/emergencies/diseases/novel-coronavirus-2019/advice-for-public?gclid=EAIaIQobChMI8PrGj-_S8AIVe9xMAh2MLwQkEAAYASAAEgLb4_D_BwE>

No vamos a divulgar sus respuestas a nadie. No pondremos sus respuestas en su expediente médico. Ud. puede no responder cualquier pregunta que le incomode. Ud. puede abandonar la encuesta en cualquier momento.

1. Ya ha recibido al menos una inyección de la vacuna contra el COVID-19?

◉ Sí ◎ No (If YES, skip to Q4. and then continue on to Q.6)

2. ¿Cree Ud. que querrá recibir la vacuna contra el COVID-19?

◉ Sí ◎ No ◎ No sé     (if YES, skip to Q.4)

3. ¿Cuál es la razón principal por la que optaría por no recibir la vacuna COVID-19?

◉ Preocupación por los tiempos apresurados

◎ Quisiera esperar para confirmar que las vacunas son seguras

◎ Desconfianza en las vacunas en general

◎ Quisiera esperar para ver qué tan efectivas son las vacunas

◎ Otro motivo ____________

4. A algunas personas les preocupa la vacunación. ¿Cree Ud. que **esas personas** aceptarían una tarjeta de regalo de $ X1 como compensación por cada inyección de la vacuna contra el COVID-19?  [Randomize amount X1 from $5 to $50 in five-dollar increments]

◉ Sí ◎ No ◎ No sé

5. Quizá Ud. puede estar preocupad@ por las vacunas. ¿Aceptaría **Ud.** una tarjeta de regalo de $ X2 como compensación por cada inyección de la vacuna contra el COVID-19? [Randomize amount X2

upwards from X1 if answer to previous question (Q4) was no; or randomize amount X2

downwards if answer to previous question (Q4) was yes.]

◉ Sí ◎ No ◎ No sé

6. Su grupo de edad:

◉ 18-29 ◎ 30-49 ◎ 50-64 ◎ 65+

7. Su género:

◉ Género no conforme / Genderqueer

◎ Hombre

◎ No binario

◎Hombre transgénero / Hombre trans

◎Mujer transgénero / Mujer trans

◎Mujer

◎No en la list (indique): ________________

◎Prefiero no contestar

8. Su grupo étnico:

◉ Negro

◎ Blanco

◎ Asiático

◎ Nativo americano

◎ Nativo de Hawái / de las islas del Pacífico

◎ Más de una raza

◎ Hispano / Latinx

◎ No hispano / latino

◎ Afroamericano

◎ Asiático Americano

◎ Otro / Preferiría no contestar

# **Supplementary Information – File 2**

## Specifications of the proportional-odds ordered logit regression models

1. Specification of the proportional-odds ordered logit (polytomous logistic) regression model used to estimate associations between respondent characteristics and the odds of other peoples’ willingness to accept conditional economic incentives for vaccination against COVID-19 **[Column 1 in Table 2]**

$$Ln\left( \frac{P\left[ y=j \right]}{1-P\left[ y=j \right]} \right)=\beta j+\beta\text{1}IncentiveAmount + \beta\text{2}VaccinationStatus+\beta\text{3}AgeGroup+\beta\text{4}Race +\beta\text{5}Female +\beta\text{6}Language + \epsilon\text{j}$$

where *j* is one of the three possible graded outcomes indicating willingness to accept (i.e., 0 = No, 1 = Unsure, 2 = Yes), and $\beta j$ is the intercept for each of these values so that P_0_[*y = j*] = e^βj^ /(1 + e^βj^). ^1,2^

2. Specification of the proportional-odds ordered logit (polytomous logistic) regression model used to estimate associations between respondent characteristics and survey respondent’s own willingness to accept conditional economic incentives to be vaccinated against COVID-19 **[Column 2 in Table 2]**

$$Ln\left( \frac{P\left[ y=j \right]}{1-P\left[ y=j \right]} \right)=\gamma j+\gamma\text{1}IncentiveAmount+\gamma\text{2}AgeGroup+\gamma\text{3}Race +\gamma\text{4}Female +\gamma\text{5}Language + \epsilon\text{j}$$

where *j* is one of the three possible graded outcomes indicating willingness to accept (i.e., 0 = No, 1 = Unsure, 2 = Yes), and$\gamma j$ is the intercept for each of these values so that P_0_[*y = j*] = e^γj^ /(1 + e^γj^) [1,2].

# **Supplementary Information – Figure 4**

**Fig. 4 Predictions of the incentive amount racial/ethnic minority adults in Rhode Island would be willing to accept for COVID-19 vaccination, by vaccination status and language of survey respondent.** Figure shows the predicted probabilities associated with the incentive amounts needed for other people to be willing-to-accept (WTA) COVID-19 vaccination stratified by select sociodemographic characteristics of the survey respondent. Vaccination status and preferred language were selected as the covariates of interest due to their statistically significant associations with odds of being WTA (Table 2). Probabilities were predicted using out-of-sample incentive amounts ranging from $50 – $250 per dose, and do not represent WTA probabilities from contingent valuation survey data. Horizontal reference lines are set at 85% based on the upper bound of most commonly cited immunization rates needed to reach herd immunity in the entire U.S. population [3,4]. Vertical lines for each incentive amount represent 95% Confidence Intervals (CI).

# **References**

1. Fautrel B, Clarke AE, Guillemin F, et al. Valuing a hypothetical cure for rheumatoid arthritis using the contingent valuation methodology: the patient perspective. *J Rheumatol*. 2005;32(3):443-453.

2. Longo A, Mitchell E, Markandya A, Galarraga I. One Size Does Not Fit All: Financial Incentives Needed to Change Physical Exercise Levels for Different Groups. *Med Decis Mak*. 2022;42(1):68-79. doi:10.1177/0272989X211011606

3. del Rio C, Malani PN, Omer SB. Confronting the Delta Variant of SARS-CoV-2, Summer 2021. JAMA. 2021;326(11):1001–1002. doi:10.1001/jama.2021.14811

4. Barker P, et al. Rethinking Herd Immunity: Managing the Covid-19 Pandemic in a Dynamic Biological and Behavioral Environment. NEJM Catalyst Innovations in Care Delivery. 2021;2(5). https://catalyst.nejm.org/doi/full/10.1056/CAT.21.0288
